# Strong couplings of scalar heavy mesons with light axial vector and pseudoscalar states in LCSR

**DOI:** 10.1016/j.heliyon.2024.e26417

**Published:** 2024-02-20

**Authors:** S. Momeni, M. Saghebfar

**Affiliations:** a Department of Physics, Isfahan University, Iran; b Optics-Laser Science and Technology Research Center, Malek Ashtar University of Technology, Isfahan, Iran

## Abstract

This study focuses on estimating the strong coupling constants of charmed and bottom mesons, such as $B^{⁎}$, $D^{⁎}$, $B_{1}$, and $D_{1}$, in relation to light pseudoscalar and axial vector states including $\pi^{-}$, $K^{-}$, $a_{1}^{-}$, $b_{1}^{-}$, $K_{1}(1270)$, and $K_{1}(1400)$. We will utilize the framework of light-cone QCD sum rules to achieve this. By employing this methodology, we will determine the values of these coupling constants and compare them to predictions from other approaches. This comparison will enable us to evaluate the accuracy and reliability of our estimations and determine the level of agreement between various theoretical models. Ultimately, our research will enhance our understanding of the strong interactions involving charmed and bottom mesons, as well as the associated light pseudoscalar and axial vector states.

## 1 Introduction

In hadron physics, the mesons which contain either a bottom or charm quark (or antiquark), are classified in the category of heavy mesons. The study of these states encompasses a wide range of aspects in high-energy physics. Various theoretical frameworks, such as lattice QCD (LQCD) [1], [2], [3], [4], [5], [6], [7], [8], [9], [10], QCD sum rules (QCDSR) [11], [12], [13], [14], [15], [16], [17], [18], [19], [20], [21], [22], [23], [24], [25], [26], [27], [28], [29], [30], [31], [32], [33], [34], [35], [36], [37], [38], Bethe-Salpeter equation [39], [40], relativistic potential model [41], [42], [43], field-correlator method (FCM) [44], light-front quark model (LFQM) [45], [46], [47], chiral extrapolation [48], extended chiral-quark model (ECQM) [49], [50], constituent quark model (CQM) [51], light-front holography [52], [53], [54], [55], [56], [57], [58], [59], [60], [61], [62], [63], [64], [65], [66], [67], [68], [69], [70], [71], [72], [73], [74], [75], hard wall AdS/QCD [76], [77], [78], [79], [80], [81], [82], [83], [84], [85], [86], etc., are employed to estimate the decay constant and mass spectroscopy of these mesons. The usefulness of the semileptonic and non-leptonic decays of this class of mesons extends to testing the Standard Model (SM) and searching for potential new physics (NP), as well as exploring the relevant Cabbibo-Kobayashi-Maskawa (CKM) matrix elements and CP violations. Additionally, the study of strong coupling constants between heavy and light mesons serves as a valuable tool for investigating final state interactions. Furthermore, the involvement of heavy mesons in vertices plays a crucial role in explaining the production and absorption cross-sections of charmonium states in heavy-ion collisions. The study of strong coupling constants also offers a unique opportunity to explore physics at large distances, far beyond the perturbative region of QCD. The QCD sum rules approach, which incorporates nonperturbative effects, holds a special position among frameworks that consider QCD at low energy scales. The QCD sum Rules investigation considers two models:

• The three-point sum rules (3PSR) model, where the correlation function is calculated in terms of QCD degrees of freedom using the Wilson operator product expansion (OPE). The vertices investigated in this model include $D_{s}^{⁎}D^{⁎}K$, $D_{s1}D_{1}K^{⁎}$ [87], $D^{⁎}D^{⁎}\rho$ [88], $D^{⁎}D\pi$, $B^{⁎}B\pi$ [89], [90], *DDρ* [91], $D^{⁎}D\rho$ [92], DDJ/ψ [93], $D^{⁎}DJ/\psi$ [94], $D^{⁎}D^{⁎}\pi$ [95], $D_{s}D^{⁎}K$, $D_{s}^{⁎}DK$ [96], *DDω* [97], $D_{s1}D^{⁎}K$, $D_{s1}D^{⁎}K_{0}^{⁎}$ [98], [99], $D_{s}D_{s}V$, $D_{s}^{⁎}D_{s}^{⁎}V$, $D_{s0}^{⁎}D_{s1}V$, $D_{s}D_{s}^{⁎}V$ [100], [101], $D_{1}D^{⁎}\pi$, $D_{1}D_{0}\pi$, $D_{1}D_{1}\pi$, $B_{1}B^{⁎}\pi$, $B_{1}B_{0}\pi$, $B_{1}B_{1}\pi$ [102], $D^{⁎}D^{⁎}J/\psi$ [103], $B_{s0}BK$ [104], $B_{s}^{⁎}BK$ [105], $D_{s}^{⁎}D_{s}\phi$ [106], $D_{s}DK_{0}^{⁎}$, $B_{s}BK_{0}^{⁎}$, $D_{s}^{⁎}DK$, $B_{s}^{⁎}BK$, $D_{s}^{⁎}DK_{1}$, $B_{s}^{⁎}BK_{1}$ [107], $D^{⁎}D\pi$, $D^{⁎}D^{⁎}\pi$, *DDρ*, $D^{⁎}D\rho$, $D^{⁎}D^{⁎}\rho$, DDJ/ψ, $D^{⁎}DJ/\psi$, $D^{⁎}D^{⁎}J/\psi$ [108], $D_{s}DK^{⁎}$, $D_{s}D^{⁎}K^{⁎}$ [109], $D_{s}^{⁎}DK^{⁎}$, $B_{s}^{⁎}BK^{⁎}$ [110], $D_{0}D_{s0}^{⁎}K^{⁎}$, $D_{1}D_{s0}^{⁎}K^{⁎}$ [111], $B_{s}B^{⁎}K$, $B_{s}BK^{⁎}$ [112], $B^{⁎}B^{⁎}\rho$ [113], $B_{s1}B^{⁎}K$, $B_{s1}B^{⁎}K_{0}^{⁎}$ [114], $B_{s1}^{⁎}B^{⁎}K$ [115], $D_{s}D_{s}J/\psi$, $D_{s}D_{s}\phi$ [116], $D^{⁎}D_{s}^{⁎}K$, $D_{1}D_{s1}K$, $D^{⁎}D_{s}K$, $D_{1}D_{s0}^{⁎}K$ [117], and $B_{s0}B_{1}K$, $B_{s1}B_{1}K$ [118].
• The light-cone QCD sum rules (LCSR) model, where the OPE is carried out near the light-cone $x^{2}\approx 0$, and the nonperturbative hadronic matrix elements are parameterized by the light-cone distribution amplitudes (LCDAs) of increasing twist. This model is used to estimate the couplings of $D^{⁎}D_{s}K$, $D_{s}^{⁎}DK$, $D_{s0}DK$, $D_{0}D_{s}K$ [119], $D^{⁎}D^{⁎}P$, $D^{⁎}DV$, *DDV* [120], $D^{⁎}D\rho$, $B^{⁎}B\rho$ [121], $D^{⁎}D\pi$, $B^{⁎}B\pi$ [122], $D^{⁎}D^{⁎}\rho$ [123], $DDA,D^{⁎}DA$ [124] and $D^{⁎}D^{⁎}V$, $B^{⁎}B^{⁎}V$, $D_{1}D_{1}V$, $B_{1}B_{1}V$ [125] vertices.

The analysis for the strong couplings $\left(B_{0},B^{⁎},A\right)$, $\left(D_{0},D^{⁎},A\right)$, $\left(B_{0},B_{1},P\right)$, and $\left(D_{0},D_{1},P\right)$ is presented in this paper using the light-cone sum rule approach. Here, *P* is the pseudoscalar ($\pi^{-},K^{-}$) and *A* is the light P-wave axial-vector mesons ($a_{1}^{-},b_{1}^{-},K_{1A}^{-},K_{1B}^{-}$). Additionally, the relationship between the axial vector physical states $K_{1}(1270)$ and $K_{1}(1400)$ mesons and the $K_{1A}$ and $K_{1B}$ states is described in terms of the mixing angle $\theta_{K}$ as:

(1) $$K_{1}(1270)=\sin\theta_{K}\;K_{1A}+\cos\theta_{K}\;K_{1B},$$

(2) $$K_{1}(1400)=\cos\theta_{K}\;K_{1A}-\sin\theta_{K},K_{1B}.$$

Various experimental data approaches were used to estimate the mixing angle $\theta_{K}$. In Ref. [126], the result was found to be $35^{\circ }<|\theta_{K}|<55^{\circ }$, while in Ref. [127], two possible solutions were estimated: $|\theta_{K}|\approx 33^{\circ }$ and $57^{\circ }$. Additionally, in [128], the value of $\theta_{K}=-{\left(34\pm 13\right)}^{\circ }$ was predicted by analyzing $B\to K_{1}(1270)\gamma$ and $\tau \to K_{1}(1270)\;\nu_{\tau }$ data. The organization of the paper is as follows: the derivation of the light-cone sum rules for the strong couplings of scalar bottom and charm mesons with the axial vector and pseudoscalar is presented in Section 2. The numerical results for the couplings can be found in Section 3, our conclusion is provided in Section 4, and the definitions for LCDAs are included in the Appendix.

## 2 Strong coupling constants in the LCSR

In the LCSR method, the strong coupling of $\left(B_{0}^{-},B^{⁎0},a_{1}^{-}\right)$ is derived by analyzing the following correlation function:

(3) $$\Pi_{\mu }(p,q)=i\int \;d^{4}x\;e^{-iq\cdot x}\;\langle 0|T\{j^{B_{0}^{-}}(x)\;\;{j_{\mu }^{B^{⁎0}}}^{†}\;(0)\}|a_{1}^{-}(p,\varepsilon_{1})\rangle ,$$

and To determine the strong coupling of the $\left(B_{0}^{-},B_{1}^{0},\pi^{-}\right)$ vertex, the following two-point correlation function is employed for computation:

(4) $$\Pi_{\nu }^{\prime }(p^{\prime },q^{\prime })=i\int \;d^{4}x\;e^{-iq\cdot x}\;\langle 0|T\{j^{B_{0}^{-}}(x)\;\;{j_{\nu }^{B_{1}^{0}}}^{†}\;(0)\}|\pi^{-}(p^{\prime })\rangle ,$$

where, the interpolating current for $B_{0}^{-}$ meson is denoted as $j^{B_{0}^{-}}=(\overset{¯}{u}\;b)$, while for $B^{⁎0}$ meson it is represented by $j_{\mu }^{B^{⁎0}}=(\overset{¯}{d}\;\gamma_{\mu }\;b)$. Similarly, the current for $B_{1}^{0}$ meson is given by $j_{\nu }^{B_{1}^{0}}=(\overset{¯}{d}\;\gamma_{\nu }\;\gamma_{5}\;b)$. These definitions hold when the time-ordering operator, denoted as T, is present. There are two ways to calculate the correlation functions $\Pi_{\mu }$ and $\Pi_{\nu }^{\prime }$ in the LCSR framework: the physical or phenomenological representation and the QCD or theoretical approaches. The dispersion relation can be used to connect these two representations of the correlation functions and determine the strong coupling constants for the $\left(B_{0}^{-},B^{⁎0},a_{1}^{-}\right)$ and $\left(B^{-}0,B_{1}^{0},\pi^{-}\right)$ vertices. By inserting two complete sets of intermediate states with the same quantum numbers as the meson currents, the phenomenological side of these correlation functions can be derived. This allows the study of vertices in terms of hadronic parameters while isolating the higher-state contributions from the pole terms of bottom mesons. After performing the Fourier transformation, the resulting expressions for $\Pi_{\mu }$ and $\Pi_{\nu }^{\prime }$ are:

(5) $$\Pi_{\mu }(p,q)=\langle 0|j^{B_{0}^{-}}(x)|B_{0}^{-}(p+q)\rangle \;\langle B^{⁎0}(q,\varepsilon_{2})|{j_{\mu }^{B^{⁎0}}}^{†}|0\rangle \;\frac{\langle B_{0}^{-}(p+q)|B^{⁎0}(q)\;a_{1}^{-}(p,\varepsilon_{1})\rangle }{\left(m_{B_{0}^{-}}^{2}-{\left(p+q\right)}^{2})\;(m_{B^{⁎0}}^{2}-q^{2}\right)}+\cdots ,$$

(6) $$\Pi_{\nu }^{\prime }(p^{\prime },q^{\prime })=\langle 0|j^{B_{0}^{-}}(x)|B_{0}^{-}(p^{\prime }+q^{\prime })\rangle \;\langle B_{1}^{0}(q^{\prime },\varepsilon_{3})|{j_{\mu }^{B_{1}^{0}}}^{†}|0\rangle \;\frac{\langle B_{0}^{-}(p^{\prime }+q^{\prime })|B_{1}^{0}(q^{\prime },\varepsilon_{3})\;\pi^{-}(p^{\prime })\rangle }{\left(m_{B_{0}^{-}}^{2}-{\left(p^{\prime }+q^{\prime }\right)}^{2})\;(m_{B_{1}^{0}}^{2}-q^{\prime \;2}\right)}+\cdots ,$$

where, the contributions from higher states are indicated by the use of ⋯. The following relations are used for the matrix elements in these correlation functions:

(7) $$\langle 0|j^{B_{0}^{-}}(x)|B_{0}^{-}(p+q)\rangle =f_{B_{0}^{-}}\;m_{B_{0}^{-}},$$

(8) $$\langle 0|j_{\mu }^{B^{⁎0}}|B^{⁎0}(q,\varepsilon_{2})\rangle =f_{B^{⁎0}}\;m_{B^{⁎0}}\;\varepsilon_{2\mu }\;,$$

(9) $$\langle B_{0}^{-}(p+q)|B^{⁎0}(q,\varepsilon_{2})\;a_{1}^{-}(p,\varepsilon_{1}\rangle =(g_{B_{0}\;B^{⁎}\;a_{1}^{-}})\;\epsilon_{\alpha \beta \gamma \sigma }\;{\left(p+q\right)}^{\alpha }\;q^{\beta }\;\varepsilon_{2}^{\gamma }\;\varepsilon_{1}^{\sigma }$$

(10) $$\langle 0|j_{\mu }^{B_{1}^{0}}|B_{1}^{0}(q^{\prime },\varepsilon_{3})\rangle =f_{B_{1}^{0}}\;m_{B_{1}^{0}}\;\varepsilon_{3\mu },$$

(11) $$\langle B_{0}^{-}(p^{\prime }+q^{\prime })|B_{1}^{0}(q^{\prime },\varepsilon_{3})\;\pi^{-}(p^{\prime })\rangle =(g_{B_{0}\;B_{1}\;\pi^{-}})\;(\varepsilon_{3}\cdot p^{\prime }).$$

The lepton polarization vectors in these relations correspond to $a_{1}^{-}$, $B^{⁎0}$, and $B_{1}^{0}$ mesons, denoted by $\varepsilon_{1}$, $\varepsilon_{2}$, and $\varepsilon_{3}$, respectively. The coupling of the $\left(B_{0}^{-},B^{⁎0},a_{1}^{-}\right)$ vertex is represented by $g_{B_{0}\;B^{⁎}\;a_{1}^{-}}$, while the coupling of the $\left(B_{0}^{-},B_{1}^{0},\pi^{-}\right)$ vertex is represented by $g_{B_{0}\;B_{1}\;\pi^{-}}$. The decay constants and masses of the states $O=(B_{0}^{-},B^{⁎0},B_{1}^{0})$, denoted by $f_{O}$ and $m_{O}$ respectively, are given in Eqs. (7), (8), (10). In the current context, the spin sum over the 4-polarization vectors $\varepsilon_{2}$ and $\varepsilon_{3}$ is employed in the following manner:

(12) $$\sum_{s}\varepsilon_{2\mu }^{s}(q)\;\varepsilon_{2\nu }^{s⁎}(q)=g_{\mu \nu }-\frac{q_{\mu }\;q_{\nu }}{q^{2}},$$

(13) $$\sum_{\lambda }\varepsilon_{3\mu }^{\lambda }(q)\;\varepsilon_{3\nu }^{\lambda ⁎}(q)=-g_{\mu \nu }+\frac{q_{\mu }\;q_{\nu }}{q^{2}}.$$

So, the phenomenological side of the correlation functions can be obtained as:

(14) $$\Pi_{\mu }(p,q)=\frac{f_{B_{0}^{-}}\;m_{B_{0}^{-}}}{\left(m_{B_{0}^{-}}^{2}-{\left(p+q\right)}^{2}\right)}\;\frac{f_{B^{⁎0}}\;m_{B^{⁎0}}}{\left(m_{B^{⁎0}}^{2}-q^{2}\right)}\;\;(g_{B_{0}\;B^{⁎}\;a_{1}})\;\epsilon_{\mu \alpha \beta \sigma }\;{\left(p+q\right)}^{\alpha }\;q^{\beta }\;\varepsilon_{1}^{\sigma }\;,$$

(15) $$\Pi_{\nu }^{\prime }(p^{\prime },q^{\prime })=\frac{f_{B_{0}^{-}}\;m_{B_{0}^{-}}}{\left(m_{B_{0}^{-}}^{2}-{\left(p^{\prime }+q^{\prime }\right)}^{2}\right)}\;\frac{f_{B_{1}^{0}}\;m_{B_{1}^{0}}}{\left(m_{B^{⁎0}}^{2}-q^{\prime \;2}\right)}\;(g_{B_{0}\;B_{1}\;\pi^{-}})\;(-p_{\mu }+\frac{q_{\mu }(p.q)}{q^{2}}).$$

In order to calculate the LCSR for the strong couplings $g_{B_{0}\;B^{⁎}\;a_{1}^{-}}$ and $g_{B_{0}\;B_{1}\;\pi^{-}}$, the theoretical side of the correlation function must be computed. Afterwards, the same structure $\epsilon_{\mu \alpha \beta \sigma }\;{\left(p+q\right)}^{\alpha }\;q^{\beta }\;\varepsilon_{1}^{\sigma }$ for the vertex $\left(B_{0}^{-},B^{⁎0},a_{1}^{-}\right)$ and the structure $p_{\mu }^{\prime }$ for the vertex $\left(B_{0}^{-},B_{1}^{0},\pi^{-}\right)$ need to be selected and compared to the results obtained from the phenomenological part. The OPE side can be calculated by inserting the meson interpolating operators into the correlation functions of Eqs. (5), (6) and using the Wick theorem in the deep Euclidean region ($-{\left(p+q\right)}^{2}\to -\infty$, $-\;q^{2}\to -\infty$ for $\Pi_{\mu }(p,q)$ and $-{\left(p^{\prime }+q^{\prime }\right)}^{2}\to -\infty$, $-\;q^{\prime \;2}\to -\infty$ for $\Pi_{\mu }^{\prime }(p^{\prime },q^{\prime })$). After inserting the interpolating operators and contracting the *b* quark field, the following result is obtained:

(16) $$\Pi_{\mu }(p,q)=\int \;d^{4}x\;e^{-iq\cdot x}\;\langle 0|\overset{¯}{u}(x)\;S^{b}(x,0)\;\gamma_{\mu }\;d(0)\}|a_{1}^{-}(p,\varepsilon_{1})\rangle ,$$

(17) $$\Pi_{\nu }^{\prime }(p^{\prime },q^{\prime })=i\int \;d^{4}x\;e^{-iq\cdot x}\;\langle 0|\overset{¯}{u}(x)\;S^{b}(x,0)\;\gamma_{\nu }\;\gamma_{5}\;d(0)|\pi^{-}(p^{\prime })\rangle ,$$

where, $S^{b}(x,0)$ is the free propagator of the *b* quark and is defined as:

(18) $$S^{b}(x,0)=i\;\langle 0|T\;\{b(x)\;\overset{¯}{b}(0)\}|0\rangle =\int \frac{d^{4}k}{{\left(2\pi \right)}^{4}}\;e^{-ik\cdot x}\frac{\mathrm{k̸}+m_{b}}{k^{2}-m_{b}^{2}}.$$

Using the Fierz identity formula, we can write:

(19) $$q_{2\alpha }^{a}(0){\overset{¯}{q}}_{1\beta }^{b}(x)=-\frac{1}{12}\delta^{ab}{\left(\Gamma_{i}\right)}_{\alpha \beta }[{\overset{¯}{q}}_{1}(x)\Gamma_{i}q_{2}(0)],$$

where, the complete set of the Dirac matrices is denoted as $\Gamma_{i}=(I,\;\gamma_{5},\;\gamma_{\alpha },\;i\gamma_{\alpha }\gamma_{5},\;\frac{1}{\sqrt{2}}\sigma_{\alpha \beta })$. It is important to note that the matrix elements $\langle 0|\overset{¯}{u}(x)\;\Gamma_{i}\;d(0)\}|a_{1}^{-}(p,\varepsilon_{1})\rangle$ and $\langle 0|\overset{¯}{u}(x)\;\Gamma_{i}\;d(0)|\pi^{-}(p^{\prime })\rangle$ are required to calculate the theoretical side of the correlation functions. The distribution amplitudes (DAs) for these two-particle states, up to twist-4, are provided in references [129], [130], [131], [132], [133]. The two-parton chiral–even distribution amplitudes for axial vector states can be found in reference [133] as:

(20) $$\langle 0|{\overset{¯}{q}}_{\alpha }(x)q_{\delta }(0)|A(p,\varepsilon )\rangle =-\frac{i}{4}\int_{0}^{1}du\;e^{-iup\cdot x}\{f_{A}m_{A}[\mathrm{p̸}\gamma_{5}\frac{\varepsilon .x}{p.x}\Phi_{∥}(u)+(\mathrm{\varepsilon ̸}-\mathrm{p̸}\frac{\varepsilon .x}{p.x})\gamma_{5}g_{⊥}^{\left(a\right)}(u)-\mathrm{x̸}\gamma_{5}\frac{\varepsilon .x}{2{\left(p.x\right)}^{2}}m_{A}^{2}{\overset{¯}{g}}_{3}(u)+\epsilon_{\mu \nu \rho \sigma }\varepsilon^{\nu }p^{\rho }x^{\sigma }\gamma^{\mu }\frac{g_{⊥}^{\left(v\right)}(u)}{4}]+\;f_{a_{1}}^{⊥}[\frac{1}{2}(\mathrm{p̸}\mathrm{\epsilon ̸}-\mathrm{\epsilon ̸}\mathrm{p̸})\gamma_{5}\;\Phi_{⊥}(u)-\frac{1}{2}(\mathrm{p̸}\mathrm{x̸}-\mathrm{x̸}\mathrm{p̸})\gamma_{5}\frac{\epsilon .x}{{\left(p.x\right)}^{2}}m_{A}^{2}{\overset{¯}{h}}_{∥}^{\left(t\right)}(u)+{i(\epsilon .x)m_{A}^{2}\gamma_{5}\frac{h_{∥}^{\left(p\right)}(u)}{2}]\}}_{\delta \alpha },$$

(21) $$\langle 0|\overset{¯}{q}(x)\gamma_{\mu }\gamma_{5}q^{\prime }(0)|A(p,\varepsilon )\rangle =if_{A}m_{A}\int_{0}^{1}du\;e^{-iup.x}\{p_{\mu }\frac{\varepsilon .x}{p.x}\Phi_{∥}(u)+\left(\varepsilon_{\mu }-p_{\mu }\frac{\varepsilon .x}{p.x}\right)g_{⊥}^{\left(a\right)}(u)+O(x^{2})\},$$

(22) $$\langle 0|\overset{¯}{q}(x)\gamma_{\mu }q^{\prime }(0)|A(p,\varepsilon )\rangle =-if_{A}\;m_{A}\;\epsilon_{\mu \nu \rho \sigma }\varepsilon^{\nu }p^{\rho }x^{\sigma }\int_{0}^{1}du\;e^{-iu\;p.x}\{\frac{g_{⊥}^{\left(v\right)}(u)}{4}+O(x^{2})\},$$

where,

(23) $${\overset{¯}{g}}_{3}(u)=g_{3}(u)+\Phi_{∥}-2g_{⊥}^{\left(a\right)}(u),$$

(24) $${\overset{¯}{h}}_{∥}^{\left(t\right)}=h_{∥}^{\left(t\right)}-\frac{1}{2}\Phi_{⊥}(u).$$

Also, the two-parton chiral–odd distribution amplitudes are defined as:

(25) $$\langle 0|\overset{¯}{q}(x)\sigma_{\mu \nu }\gamma_{5}q^{\prime }(0)|A(p,\varepsilon )\rangle =f_{A}^{⊥}\int_{0}^{1}du\;e^{-iup^{\prime }.x}\{(\varepsilon_{\mu }p_{\nu }-\varepsilon_{\nu }p_{\mu })\Phi_{⊥}(u)+\frac{m_{A}^{2}\;\varepsilon .x}{{\left(p.x\right)}^{2}}(p_{\mu }x_{\nu }-p_{\nu }x_{\mu }){\overset{¯}{h}}_{∥}^{\left(t\right)}+O(x^{2})\},$$

(26) $$\langle 0|\overset{¯}{q}(x)\gamma_{5}q^{\prime }(0)|A(p,\varepsilon )\rangle =f_{A}^{⊥}m_{A}^{2}(\varepsilon .x)\int_{0}^{1}du\;e^{-iup.x}\{\frac{h_{∥}^{\left(p\right)}(u)}{2}+O(x^{2})\},$$

where, $A=(a_{1}^{-},b_{1}^{-},K_{1A},K_{1B})$. In Eqs. (20)-(26), $\Phi_{∥}$ and $\Phi_{⊥}$ are the twist-2, $g_{⊥}^{\left(a\right)}$, $g_{⊥}^{\left(v\right)}$, $h_{∥}^{\left(t\right)}$ and $h_{∥}^{\left(p\right)}$ are twist-3, and $g_{3}$ is twist-4 functions. Moreover, $f_{A}$ is a scale–independent decay constant and conserves *G*-parity, while $f_{A}^{⊥}$ is scale–dependent and violates *G*-parity [133]. The definitions for these LCDAs are given in Appendix.

For pseudoscalar mesons to twist-4 accuracy, the two-particle DAs are defined as [129], [130], [131], [132]:

(27) $$\langle 0|\overset{¯}{q}(x)\gamma_{\mu }\;\gamma_{5}\;q^{\prime }(0)|P(p)\rangle =i\;p_{\mu }f_{P}\int_{0}^{1}du\;e^{-iup\cdot x}\varphi_{P}(u),$$

(28) $$\langle 0|\overset{¯}{q}(x)\gamma_{\mu }\;q^{\prime }(0)|P(p)\rangle =-f_{P}\int_{0}^{1}du\;e^{-iup\cdot x}(x_{\mu }-\frac{x^{2}p_{\mu }}{p\cdot x})\;g_{2}(u),$$

(29) $$\langle 0|\overset{¯}{q}(x)i\gamma_{5}q^{\prime }(0)|P(p)\rangle =\frac{f_{P}\;m_{P}^{2}}{m_{q}+m_{q^{\prime }}}\;\int_{0}^{1}du\;e^{-iup\cdot x}\;\phi^{p}(u),$$

(30) $$\langle 0|\overset{¯}{q}(x)\sigma_{\alpha \beta }\gamma_{5}\;q^{\prime }(0)|P(P)\rangle =-\frac{i}{6}\;\frac{f_{P}\;m_{P}^{2}}{m_{q}+m_{q^{\prime }}}(p_{\alpha }\;x_{\beta }-x_{\beta }\;x_{\alpha })\int_{0}^{1}du\;e^{-iup\cdot x}\;\phi^{\sigma }(u),$$

where, P=π,K. The full expression of the corresponding DAs can be found in Appendix.

The collection of the structures $\epsilon_{\mu \alpha \beta \sigma }\;{\left(p+q\right)}^{\alpha }\;q^{\beta }\;\varepsilon_{1}^{\sigma }$ forming $\Pi_{\mu }(p,q)$ and $p_{\mu }^{\prime }$ forming $\Pi_{\nu }^{\prime }(p^{\prime },q^{\prime })$ is achieved by inserting the free propagator of *b* quark [Eq. (18)] and two-particle LCDAs [Eqs. (20)-(22), (25)-(30)] in correlation functions [Eqs. (16), (17)], followed by tracing and integrating. To calculate the strong couplings, the final step involves performing the double Borel transformation with respect to the variables $-{\left(p+q\right)}^{2}$ and $-q^{2}$ for the correlation function $\Pi_{\mu }$, and $-{\left(p^{\prime }+q^{\prime }\right)}^{2}$ and $-q^{\prime \;2}$ for the correlation function $\Pi_{\mu }^{\prime }$. The strong coupling relations are extracted using the following Borel transformations:

(31) $$B_{R^{2}}(M^{2})[\frac{1}{{\left(R^{2}-m^{2}\right)}^{n}}]=\frac{{\left(-1\right)}^{n}}{\Gamma (n)}\frac{e^{-\frac{m^{2}}{M^{2}}}}{{\left(M^{2}\right)}^{n}},$$

(32) $$B_{R^{2}}(M^{2})[e^{-\frac{R^{2}}{m^{2}}}]=\delta (1-\frac{M^{2}}{m^{2}}),$$

where parameter *M* is known as the Borel mass. In order to perform the continuum subtraction, we employ the method outlined in [131]. Consequently, the strong form factors $g_{B_{0},B^{⁎},a_{1}^{-}}$ and $g_{B_{0},B_{1},\pi^{-}}$ can be derived using the following sum rule:

(33) $$g_{B_{0}\;B^{⁎}\;a_{1}^{-}}=\frac{\Lambda (u_{0},s_{0}^{B_{0}},a_{1})\;\Theta (B_{0},B^{⁎})}{\Delta (B_{0},B^{⁎})}\;(\frac{3}{16}\;f_{a_{1}}\;m_{a_{1}}\;m_{b}\;g_{⊥}^{v}(u_{0},\mu )+\frac{5}{12}\;f_{a_{1}}^{⊥}\;M_{0}^{2}\;\Phi_{⊥}(u_{0},\mu )+\frac{1}{3}\;f_{a_{1}}^{⊥}\;m_{a_{1}}^{2}\;{\overset{¯}{h}}_{∥}^{ii(t)}),$$

(34) $$g_{B_{0}\;B_{1}\;\pi^{-}}=\frac{\Lambda (u_{0},s_{0}^{B_{0}},\pi )\;\Theta (B_{0},B_{1})\;f_{\pi }}{\Delta (B_{0},B_{1})}(-\frac{4\;\mu_{\pi }}{3}\;\phi^{\sigma }(u_{0},\mu )\;\left[\delta_{1}(u_{0})-2\;m_{\pi }^{2}\;\delta_{2}(u_{0}\right)]-\frac{8\;\mu_{\pi }\;u_{0}\;M_{0}^{4}}{3\;M_{1}^{2}}\;(\partial_{u}\phi^{\sigma }(u,\mu )){|}_{u=u_{0}}))+\frac{\Lambda (u_{0},s_{0}^{B_{0}},\pi )\;\Theta (B_{0},B_{1})\;f_{\pi }}{\Delta (B_{0},B_{1})}(\frac{\mu_{\pi }\;M_{0}^{4}}{3\;M_{1}^{2}}\;\phi^{p}(u_{0},\mu )\;+m_{b}\;M_{0}^{2}\;\phi_{\pi }(u_{0},\mu )\;+\frac{2\;m_{b}}{3}\;g_{2}(u_{0},\mu )),$$

where,

(35) $$u_{0}=\frac{M_{2}^{2}}{M_{1}^{2}+M_{2}^{2}},\;\;\;\;\;M_{0}^{2}=\frac{M_{1}^{2}\;M_{2}^{2}}{M_{1}^{2}+M_{2}^{2}},\;\;\;\;\;\mu_{\pi }=\frac{m_{\pi }^{2}}{m_{u}+m_{d}},$$

(36) $$\Lambda (u_{0},s_{0}^{B},O)=\exp\left[-\frac{u_{0}\;(1-u_{0})\;m_{O}^{2}+m_{b}^{2}}{M_{0}^{2}}\right]-\exp\left[-\frac{s_{0}^{B}}{M_{0}^{2}}\right],$$

(37) $$\Delta (O_{1},O_{2})=\left(f_{O_{1}}\;m_{O_{1}}\right)\;\left(f_{O_{2}}\;m_{O_{2}}\right),$$

(38) $$\Theta (O_{1},O_{2})=\exp\left[\frac{m_{O_{1}}^{2}}{M_{1}^{2}}+\frac{m_{O_{2}}^{2}}{M_{2}^{2}}\right],$$

(39) $$\delta_{1}(u_{0})=M_{0}^{2}+\frac{1}{2}\;m_{b}-\frac{2\;M_{0}^{4}}{M_{1}^{2}}\;,$$

(40) $$\delta_{2}(u_{0})=u_{0}^{2}\;+\;\frac{2\;M_{0}^{2}}{M_{1}^{2}}\;(2\;u_{0}-1),$$

and $s_{0}^{B_{0}}$ is threshold parameter for scalar *B* meson. Moreover, $M_{1}^{2}\;(M_{2}^{2})$ in Eq. (33) and Eq. (34), is the Borel parameter with respect to $-{\left(p+q\right)}^{2}(-q^{2})$ and $-{\left(p^{\prime }+q^{\prime }\right)}^{2}(-q^{\prime \;2})$ in $\Pi_{\mu }$ and $\Pi_{\mu }^{\prime }$.

In order to obtain $g_{B_{0}\;B^{⁎}\;b_{1}^{-}}$ in Equation (33), we need to apply the substitution $a_{1}^{-}\to b_{1}^{-}$, and for the couplings of the $\left(B_{s0},B^{⁎-},K_{1A(B)}^{-}\right)$ vertex in Equation (33), the following changes need to be made: $B_{0}\to B_{s0}$, $B^{⁎}\to B^{⁎-}$, $a_{1}^{-}\to K_{1A(B)}^{-}$ and $m_{u(d)}\to m_{s}$. Furthermore, by replacing $B_{0}\to B_{s0}$, $B_{1}\to B_{1}^{-}$, $\pi^{-}\to K^{-}$ and $m_{u(d)}\to m_{s}$, the coupling of the $\left(B_{s0},B_{1}^{-},K^{-}\right)$ vertex can be derived from Equation (34). Additionally, to calculate the coupling of the $\left(D_{0}^{-},{\overset{¯}{D}}^{⁎0},a_{1}^{-}\right)$ and $\left(D_{0}^{-},{\overset{¯}{D}}_{1}^{0},\pi^{-}\right)$ vertices, we substitute $B_{0}\to D_{0}$, $B^{⁎}\to D^{⁎}$, $m_{b}\to m_{c}$ and $s_{0}^{B_{0}}\to s_{0}^{D_{0}}$.

## 3 Numerical analysis

In this section, we provide a numerical analysis of the LSCR predictions for the couplings of $\left(B_{0},B^{⁎},A\right)$, $\left(D_{0},D^{⁎},A\right)$, $\left(B_{0},B_{1},P\right)$, and $\left(D_{0},D_{1},P\right)$ vertices, where $A=(a_{1}^{-},b_{1}^{-},K_{1A}^{-},K_{1B}^{-})$ and $P=(\pi^{-},K^{-})$. The particle masses, decay constants, and continuum threshold parameters considered in our analysis are shown in Table 1. The values given in Table 1 for the scalar, vector, and pseudoscalar bottom and charm mesons are obtained using the QCDSR framework, which incorporates calculations of the vacuum condensates up to dimension-6 and includes the $O(\alpha_{s})$ corrections to the quark condensates in [38]. Additionally, the values of $f_{A}$ for $A=(a_{1}^{-},b_{1}^{-},K_{1A}^{-},K_{1B}^{-})$ and the masses of $K_{1A}^{-}$ and $K_{1B}^{-}$ are taken from the QCDSR of [134]. Experimental data of [135] provide the other mentioned values in Table 1, while the chosen continuum threshold parameters are obtained from [38] and are close to the mass of the first excited state with the same quantum numbers as the interpolating currents in question. To determine the factorization scale *μ* for input parameters of DAs in our numeric analysis, we use different values depending on the vertices involved in bottom mesons and other vertices. Specifically, for the vertices involving bottom mesons, the scale $\mu_{b}$ is calculated as $\mu_{b}={\left(m_{B_{0}}^{2}-m_{b}^{2}\right)}^{\frac{1}{2}}≃2.2\;\mathrm{GeV}$. On the other hand, for vertices involving $\left(D_{0},D^{⁎},A\right)$ and $\left(D_{0},D_{1},P\right)$, the scale $\mu_{c}$ is fixed as $\mu_{c}={\left(m_{D_{0}}^{2}-m_{c}^{2}\right)}^{\frac{1}{2}}≃1\;\mathrm{GeV}$. We use these two scales for the corresponding vertices, while the other input parameters remain the same. The Gegenbauer moments of the twist-2 and twist-3 LCDAs of axial-vector mesons for these two scales are provided in Table 2 from [134]. For the scale-dependent decay constant of axial vector states ($f_{A}^{⊥}$), we define $f_{A}=f_{A}^{⊥}$ at μ=1GeV according to [134]. To obtain this parameter at $\mu =\mu_{b}$, we refer to [134]:

(41) $$(f_{A}^{⊥}a_{l}^{⊥,A})(\mu_{b})=(f_{A}^{⊥}a_{l}^{⊥,A})(\mu_{c}){\left(\frac{\alpha_{s}(\mu_{c})}{\alpha_{s}(\mu_{b})}\right)}^{-\gamma_{\left(l\right)}^{⊥}/b}\;,$$

where $b=(11N_{c}-2n_{f})/3$, and $\gamma_{\left(l\right)}^{⊥}$ is the one-loop anomalous dimensions and is defined as [136], [137]:

(42) $$\gamma_{\left(l\right)}^{⊥}=C_{F}\left(1+4\sum_{j=2}^{l+1}\frac{1}{j}\right).$$

$C_{F}$ in this equation is represented as $C_{F}=(N_{c}^{2}-1)/(2N_{c})$. We utilize the values $\alpha_{s}(\mu_{c})=0.495$ and $\alpha_{s}(\mu_{b})=0.287$ [133], [135] for the strong coupling $\alpha_{s}(\mu )$ in our numerical analysis. The input values of $a_{1}$, $a_{2}$, $f_{3}$, $f_{3}$, $\omega_{3}$, $\lambda_{3}$ and $\delta^{2}$ for DAs of $\pi^{-}$ and $K^{-}$ are presented in Table 3. Additionally, this table includes the values of quark masses at scales $\mu =(\mu_{b},\mu_{c})$, which are taken from [138].

**Table 1:** Masses, decay constants and continuum threshold parameters of the heavy and light mesons. The values of heavy bottom and charm mesons, decay constant of axial vectors $\left(a_{1}^{-},b_{1}^{-},K_{1A}^{-},K_{1B}^{-}\right)$, $m_{K_{1A}}$ and $m_{K_{1B}}$ are calculated with QCDSR method [38], [134]. The masses of $a_{1}^{-}$, $b_{1}^{-}$, *π*^−^ and *K*^−^ as well as *f*_*π*_ and *f*_*K*_ are taken from experimental data [135], and the values of *s*_0_ can be found in [38].

	Mass (GeV)	Decay constant (MeV)	$s_{0}(\mathrm{Ge}V^{2})$
*D*^⁎^	2.01 ± 0.08	263 ± 21	−−
*D*_0_	2.40 ± 0.05	373 ± 19	8.3 ± 0.5
*D*_*s*0_	2.32 ± 0.05	333 ± 20	7.4 ± 0.5
*D*_1_	2.42 ± 0.05	332 ± 18	−−
*B*^⁎^	5.32 ± 0.06	213 ± 18	−−
*B*_0_	5.72 ± 0.05	281 ± 14	40.0 ± 1.0
*B*_*s*0_	5.70 ± 0.06	274 ± 13	40.0 ± 1.0
*B*_1_	5.74 ± 0.05	335 ± 18	−−
$a_{1}^{-}$	1.23 ± 0.06	238 ± 10	−−
$b_{1}^{-}$	1.21 ± 0.07	180 ± 8	−−
$K_{1A}^{-}$	1.31 ± 0.06	250 ± 13	−−
$K_{1B}^{-}$	1.34 ± 0.08	190 ± 10	−−
*π*^−^	0.14 ± 0.00	130.4	−−
*K*^−^	0.49 ± 0.00	155.5	−−

**Table 2:** Gegenbauer moments of twist-2 and twist-3 LCDAs of the axial vector mesons $A=(a_{1}^{-},b_{1}^{-},K_{1A},K_{1B})$ from [133].

*μ*	$\mu_{c}=1\;(\mathrm{GeV})$			
Axial vector (A)	$a_{1}^{-}$	$b_{1}^{-}$	*K*_1*A*_	*K*_1*B*_
$a_{0}^{⊥}$	0	1	0.08 ± 0.09	1
$a_{1}^{⊥}$	−1.04 ± 0.34	0	−1.08 ± 0.48	0.17 ± 0.22
$a_{2}^{⊥}$	0	0.03 ± 0.19	0.02 ± 0.20	−0.02 ± 0.22
$a_{0}^{∥}$	1	0	1	0.14 ± 0.15
$a_{1}^{∥}$	0	−1.95 ± 0.35	0	−1.95 ± 0.45
$a_{2}^{∥}$	−0.02 ± 0.02	0	−0.05 ± 0.03	0.02 ± 0.10
$f_{3}^{V}{\left(\mathrm{Gev}\right)}^{2}$	0.0055 ± 0.0027	0.0052 ± 0.0018	0.0052 ± 0.0027	0.0049 ± 0.0021
*ω*^*V*^	−2.9 ± 0.9	−1.5 ± 0.4	−3.1 ± 1.1	−1.9 ± 0.6
$f_{3}^{A}{\left(\mathrm{Gev}\right)}^{2}$	0.0022 ± 0.0009	−0.0058 ± 0.0023	0.0026 ± 0.0013	−0.0065 ± 0.0029
*σ*^*V*^	0	0	−0.13 ± 0.16	0.35 ± 0.73
*λ*^*A*^	0	0	0.57 ± 0.39	0.07 ± 0.19
*σ*^*A*^	0	0	2.4 ± 2.0	−0.06 ± 0.05



*μ*	$\mu_{b}=2.2\;(\mathrm{GeV})$			
Axial vector (A)	$a_{1}^{-}$	$b_{1}^{-}$	*K*_1*A*_	*K*_1*B*_
$a_{0}^{⊥}$	0	1	0.07 ± 0.08	1
$a_{1}^{⊥}$	−0.85 ± 0.28	0	−0.88 ± 0.39	0.14 ± 0.18
$a_{2}^{⊥}$	0	0.02 ± 0.15	0.01 ± 0.15	−0.02 ± 0.17
$a_{0}^{∥}$	1	0	1	0.14 ± 0.15
$a_{1}^{∥}$	0	−1.61 ± 0.29	0	−1.61 ± 0.37
$a_{2}^{∥}$	−0.01 ± 0.01	0	−0.04 ± 0.02	0.01 ± 0.07
$f_{3}^{V}{\left(\mathrm{Gev}\right)}^{2}$	0.0036 ± 0.0018	0.0030 ± 0.0011	0.0034 ± 0.0018	0.0029 ± 0.0012
*ω*^*V*^	−2.9 ± 0.9	−1.4 ± 0.3	−3.1 ± 1.1	−1.7 ± 0.4
$f_{3}^{A}{\left(\mathrm{Gev}\right)}^{2}$	0.0012 ± 0.0005	−0.0036 ± 0.0014	0.0014 ± 0.0007	−0.0041 ± 0.0018
*σ*^*V*^	0	0	−0.13 ± 0.16	0.31 ± 0.68
*λ*^*A*^	0	0	0.70 ± 0.46	0.09 ± 0.24
*σ*^*A*^	0	0	2.4 ± 2.0	−0.05 ± 0.04

**Table 3:** Hadronic parameters for the *π*^−^ and *K*^−^ DAs as well as the masses of quarks at $\mu_{c}=1\;(\mathrm{GeV})$ and $\mu_{b}≃2.2\;(\mathrm{GeV})$ scales from [138].

*μ*	$\mu_{c}$		$\mu_{b}$	
Pseudoscalar (P)	*π*^−^	*K*^−^	*π*^−^	*K*^−^

*a*_1_	0	0.06 ± 0.03	0	0.05 ± 0.02
*a*_2_	0.17 ± 0.10	0.17 ± 0.10	0.25 ± 0.15	0.25 ± 0.15
*a*_>3_	0	0	0	0
$f_{3}\times 10^{2}\;(\mathrm{Ge}V^{2})$	0.45 ± 0.15	0.45 ± 0.15	0.31 ± 0.10	0.33 ± 0.11
*ω*_3_	−1.50 ± 0.70	−1.20 ± 0.70	−1.10 ± 0.50	−0.90 ± 0.50
*λ*_3_	0	1.60 ± 0.40	0	1.45 ± 0.35
$\delta^{2}\;(\mathrm{Ge}V^{2})$	0.18 ± 0.06	0.20 ± 0.06	0.14 ± 0.05	0.17 ± 0.05
${\overset{¯}{m}}_{u,d\;(s)}(\mathrm{MeV})$	5.6 ± 1.6	(137 ± 2)	4.1 ± 1.1	(100 ± 20)

The couplings of the vertices $\left(B_{0}^{-},B^{⁎0},a_{1}^{-}\right)$, $\left(B_{0}^{-},B^{⁎0},b_{1}^{-}\right)$, $\left(B_{s0},B^{⁎-},K_{1A}^{-}\right)$, $\left(B_{s0},B^{⁎-},K_{1B}^{-}\right)$, $\left(B_{0}^{-},B_{1}^{0},\pi^{-}\right)$, $\left(B_{s0},B_{1}^{-},K^{-}\right)$, $\left(D_{0}^{-},{\overset{¯}{D}}^{⁎0},a_{1}^{-}\right)$, $\left(D_{0}^{-},{\overset{¯}{D}}^{⁎0},b_{1}^{-}\right)$, $\left(D_{s0}^{-},{\overset{¯}{D}}^{⁎0},K_{1A}^{-}\right)$, $\left(D_{s0}^{-},{\overset{¯}{D}}^{⁎0},K_{1B}^{-}\right)$, $\left(D_{0}^{-},{\overset{¯}{D}}_{1}^{0},\pi^{-}\right)$, and $\left(D_{s0}^{-},{\overset{¯}{D}}_{1}^{0},K^{-}\right)$ can now be estimated since we have all the input parameters. It is important to note that two auxiliary parameters $M_{1}^{2}$ and $M_{2}^{2}$, which are the Borel mass parameters, are present in Eqs. (33), (34). Additionally, it is worth mentioning that the initial and final heavy mesons have identical or very similar masses, establishing the following relations:

(43) $$\frac{m_{B_{0}}}{m_{B_{0}}+m_{B_{\left(1\right)}^{⁎}}}=0.51(0.49),\;\;\;\;\;\;\;\;\;\;\;\;\frac{m_{B_{s0}}}{m_{B_{s0}}+m_{B_{\left(1\right)}^{⁎}}}=0.51(0.50),$$

(44) $$\frac{m_{D_{0}}}{m_{D_{0}}+m_{D_{\left(1\right)}^{⁎}}}=0.51(0.49),\;\;\;\;\;\;\;\;\;\;\;\;\frac{m_{D_{s0}}}{m_{D_{s0}}+m_{D_{\left(1\right)}^{⁎}}}=0.52(0.49).$$

**Figure 1:**
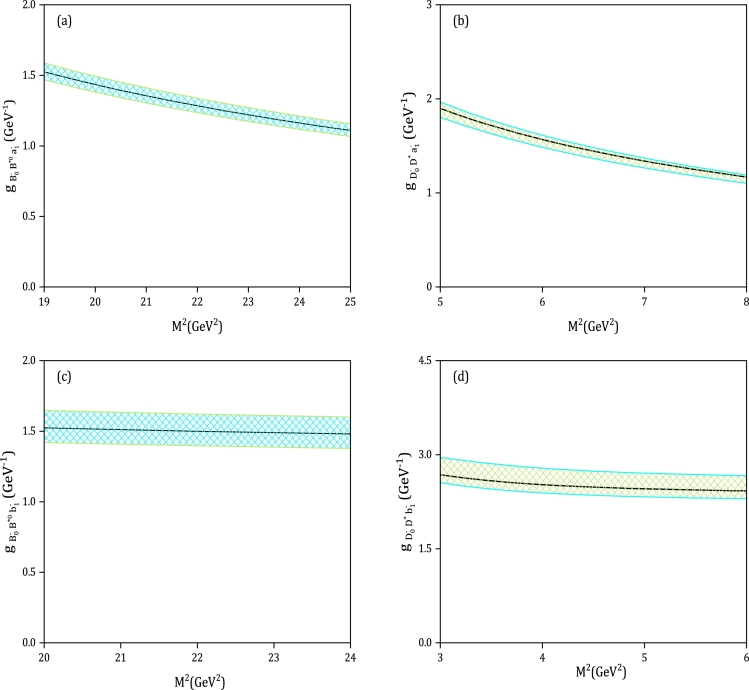
Dependence of the coupling constant of the vertex $\left(B_{0}^{-},B^{⁎0},a_{1}^{-}\right)$ (part (a)), $\left(D_{0}^{-},{\overset{¯}{D}}^{⁎0},a_{1}^{-}\right)$ (part (b)), $\left(B_{0}^{-},B^{⁎0},b_{1}^{-}\right)$ (part (c)), and $\left(D_{0}^{-},{\overset{¯}{D}}^{⁎0},b_{1}^{-}\right)$ (part (d)) on *M*^2^ with their uncertainty regions.

**Figure 2:**
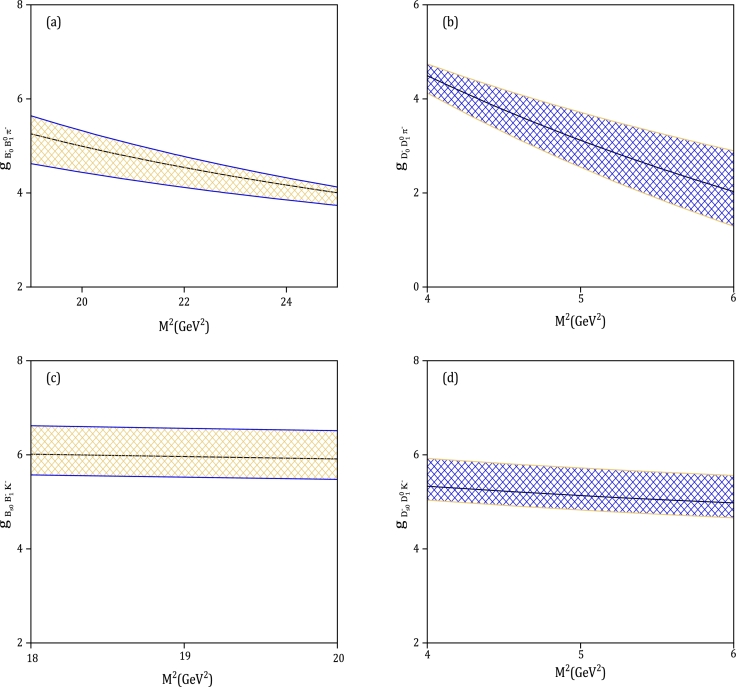
Strong form factors of $\left(B_{0}^{-},B_{1}^{0},\pi^{-}\right)$ (part (a)), $\left(D_{0}^{-},{\overset{¯}{D}}_{1}^{0},\pi^{-}\right)$ (part (b)), $\left(B_{s0},B_{1}^{-},K^{-}\right)$ (part (c)), and $\left(D_{s0}^{-},{\overset{¯}{D}}_{1}^{0},K^{-}\right)$ (part (d)) vertices as functions of the Borel mass parameter, *M*^2^, with their uncertainty regions.

Hence, it is convenient to choose $M_{1}^{2}=M_{2}^{2}=2\;M^{2}$ as the value. Consequently, our task is simplified to determining the active region of the $M^{2}$ parameter. It is important to note that the contributions from higher twist functions should be significantly smaller than the leading-twist terms, which sets the lower limit of the Borel mass $M^{2}$. To satisfy this requirement, the Borel mass region should be selected in such a way that the strong coupling constants remain relatively stable. Conversely, the upper limit of this parameter is determined by minimizing the impact of the continuum and higher states. By considering these two conditions, we can obtain the intervals of $M^{2}$ as shown in Table 4. Figs. 1(a, b, c, d) plot the dependence of the strong coupling constant of $\left(B_{0}^{-},B^{⁎0},a_{1}^{-}\right)$, $\left(D_{0}^{-},{\overset{¯}{D}}^{⁎0},a_{1}^{-}\right)$, $\left(B_{0}^{-},B^{⁎0},b_{1}^{-}\right)$, and $\left(D_{0}^{-},{\overset{¯}{D}}^{⁎0},b_{1}^{-}\right)$ vertices on the Borel mass parameter, $M^{2}$. The solid lines represent the calculated LCSR estimation for the couplings, while the shaded regions take into account their errors. Fig. 2 (a, b, c, d) displays the central values and uncertainty regions for the couplings of $\left(B_{0}^{-},B_{1}^{0},\pi^{-}\right)$, $\left(D_{0}^{-},{\overset{¯}{D}}_{1}^{0},\pi^{-}\right)$, $\left(B_{s0},B_{1}^{-},K^{-}\right)$, and $\left(D_{s0}^{-},{\overset{¯}{D}}_{1}^{0},K^{-}\right)$ vertices as a function of $M^{2}$. Furthermore, Table 5 presents the estimated values for the coupling constants of $\left(B_{0},B^{⁎},A\right)$ and $\left(B_{0},B_{1},P\right)$ vertices. The variation of masses of heavy mesons, decay constants, and the Gegenbauer moments of twist-2 LCDAs, as shown in Figure 1, Figure 2, and Table 5, is the main factor contributing to uncertainties. Twist-2 amplitudes ($\Phi_{⊥}^{A},\phi_{P}$) account for about 77%−89% of the total value of the vertices, including charm mesons. For the couplings of scalar *B* mesons, this contribution ranges from 80% to 87%. In our investigation, we utilize the formalism introduced in [139] to incorporate next-to-leading-order (NLO) twist-2 and twist-3 terms. Our numerical analysis reveals that approximately 2.7% of the total value of $g_{B^{-}0B1^{0}\pi^{-}}$ can be attributed to NLO corrections in twist-3 terms. Additionally, the contribution from NLO twist-2 terms is 8.02% of the total value.

**Table 4:** Working region of the Borel mass parameter for (*B*_0_,*B*^⁎^,*A*) and (*B*_0_,*B*_1_,*P*) vertices.

Vertex	$M^{2}\;\left(\mathrm{Ge}V^{2}\right)$	Vertex	$M^{2}\;\left(\mathrm{Ge}V^{2}\right)$
$\left(B_{0}^{-},B^{⁎0},a_{1}^{-}\right)$	19.0 ≤ *M*^2^ ≤ 25.0	$\left(D_{0}^{-},{\overset{¯}{D}}^{⁎0},a_{1}^{-}\right)$	5.0 ≤ *M*^2^ ≤ 8.0
$\left(B_{0}^{-},B^{⁎0},b_{1}^{-}\right)$	20.0 ≤ *M*^2^ ≤ 24.0	$\left(D_{0}^{-},{\overset{¯}{D}}^{⁎0},b_{1}^{-}\right)$	3.0 ≤ *M*^2^ ≤ 6.0
$\left(B_{s0},B^{⁎-},K_{1A}^{-}\right)$	19.0 ≤ *M*^2^ ≤ 24.0	$\left(D_{s0}^{-},{\overset{¯}{D}}^{⁎0},K_{1A}^{-}\right)$	4.0 ≤ *M*^2^ ≤ 7.0
$\left(B_{s0},B^{⁎-},K_{1B}^{-}\right)$	20.0 ≤ *M*^2^ ≤ 25.0	$\left(D_{s0}^{-},{\overset{¯}{D}}^{⁎0},K_{1B}^{-}\right)$	3.0 ≤ *M*^2^ ≤ 7.0
$\left(B_{0}^{-},B_{1}^{0},\pi^{-}\right)$	19.0 ≤ *M*^2^ ≤ 25.0	$\left(D_{0}^{-},{\overset{¯}{D}}_{1}^{0},\pi^{-}\right)$	4.0 ≤ *M*^2^ ≤ 6.0
$\left(B_{s0},B_{1}^{-},K^{-}\right)$	18.0 ≤ *M*^2^ ≤ 20.0	$\left(D_{s0}^{-},{\overset{¯}{D}}_{1}^{0},K^{-}\right)$	4.0 ≤ *M*^2^ ≤ 6.0

**Table 5:** Values of the strong coupling constants of scalar *B* and *D* mesons with light axial vector and pseudoscalar mesons. Predictions of 3PSR [102], [117] are also presented for completeness.

Vertex	3PSR [102]	This work	Vertex	3PSR [102], [117]	This work
$\left(B_{0}^{-},B^{⁎0},a_{1}^{-}\right)$	−	1.37 ± 0.21	$\left(D_{0}^{-},{\overset{¯}{D}}^{⁎0},a_{1}^{-}\right)$	−	1.49 ± 0.52
$\left(B_{0}^{-},B^{⁎0},b_{1}^{-}\right)$	−	1.51 ± 0.21	$\left(D_{0}^{-},{\overset{¯}{D}}^{⁎0},b_{1}^{-}\right)$	−	1.82 ± 0.46
$\left(B_{s0},B^{⁎-},K_{1A}^{-}\right)$	−	1.72 ± 0.27	$\left(D_{s0}^{-},{\overset{¯}{D}}^{⁎0},K_{1A}^{-}\right)$	−	2.15 ± 0.31
$\left(B_{s0},B^{⁎-},K_{1B}^{-}\right)$	−	2.45 ± 0.13	$\left(D_{s0}^{-},{\overset{¯}{D}}^{⁎0},K_{1B}^{-}\right)$	−	3.58 ± 0.53
$\left(B_{0}^{-},B_{1}^{0},\pi^{-}\right)$	4.68 ± 1.44	4.62± 0.72	$\left(D_{0}^{-},{\overset{¯}{D}}_{1}^{0},\pi^{-}\right)$	3.92 ± 0.93	3.80 ± 0.81
$\left(B_{s0},B_{1}^{-},K^{-}\right)$	−	6.01 ± 0.52	$\left(D_{s0}^{-},{\overset{¯}{D}}_{1}^{0},K^{-}\right)$	4.90 ± 1.91	5.33 ± 0.62

The couplings of scalar bottom and charm mesons with the physical states $K_{1}(1270)$ and $K_{1}(1400)$ are obtained using the following relations:

(45) $$g^{K_{1}(1270)}=\sin\theta_{K}\;g^{K_{1A}}+\cos\theta_{K}\;g^{K_{1B}},$$

(46) $$g^{K_{1}(1400)}=\cos\theta_{K}\;g^{K_{1A}}-\sin\theta_{K}\;g^{K_{1B}}.$$

**Figure 3:**
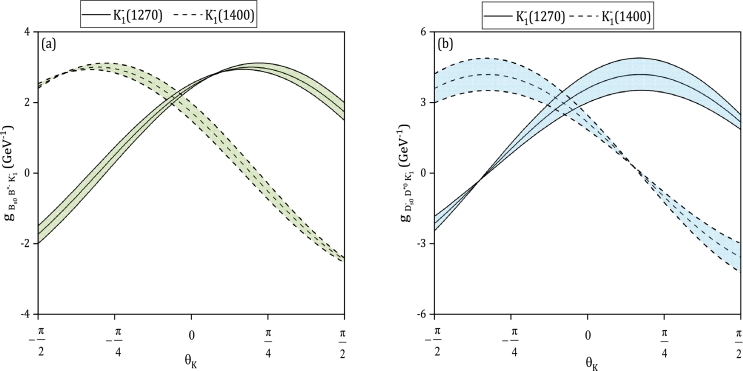
Strong form factors of $\left(B_{s0},B^{⁎-},K_{1}^{-}\right)$ (part (a)), and $\left(D_{s0}^{-},{\overset{¯}{D}}^{⁎0},K_{1}^{-}\right)$ (part (b)) vertices with *K*_1_ = (*K*_1_(1270),*K*_1_(1400)) as functions of the mixing angle *θ*_*K*_.

The couplings of $\left(B_{s0},B^{⁎-},K_{1}^{-}\right)$ and $\left(D_{s0}^{-},{\overset{¯}{D}}^{⁎0},K_{1}^{-}\right)$ vertices, denoted by $g^{K_{1}}$ with $K_{1}=(K_{1A},K_{1B},K_{1}(1270),K_{1}(1400))$, are shown in Fig. 3 (a,b) depicting the dependence on $\theta_{K}$. The figure displays the solid lines representing the couplings of scalar *B* and *D* mesons with $K_{1}^{-}(1270)$, while the dashed lines represent the couplings with $K_{1}^{-}(1400)$. The uncertainty regions are also included in the figure. In Table 6, the values of the strong couplings of vertices $\left(B_{s0},B^{⁎-},K_{1}^{-}(1270)\right)$, $\left(B_{s0},B^{⁎-},K_{1}^{-}(1400)\right)$, $\left(D_{s0}^{-},{\overset{¯}{D}}^{⁎0},K_{1}^{-}(1270)\right)$, and $\left(D_{s0}^{-},{\overset{¯}{D}}^{⁎0},K_{1}^{-}(1400)\right)$ at $\theta_{K}=-{\left(34\pm 13\right)}^{\circ }$ [128] and $|\theta_{K}|\approx 57^{\circ }$ [127] represent the final and last important point.

**Table 6:** Values of the strong coupling constants of $\left(B_{s0},B^{⁎-},K_{1}^{-}\right)$ and $\left(D_{s0}^{-},{\overset{¯}{D}}^{⁎0},K_{1}^{-}\right)$ with *K*_1_ = (*K*_1_(1270),*K*_1_(1400)) vertices in (GeV^−1^), at the various mixing angles.

*θ*_*K*_	−57^∘^	−47^∘^	−34^∘^	−21^∘^	57^∘^
$g_{B_{s0}B^{⁎-}K_{1}^{-}(1270)}$	−0.11 ± 0.24	0.45 ± 0.23	1.01 ± 0.16	1.69 ± 0.15	2.70 ± 0.12
$g_{B_{s0}B^{⁎-}K_{1}^{-}(1400)}$	2.99 ± 0.05	2.97 ± 0.11	2.80 ± 0.17	2.47 ± 0.20	−1.09 ± 0.16

$g_{D_{s0}^{-}{\overset{¯}{D}}^{⁎0}K_{1}^{-}(1270)}$	−0.14 ± 0.05	0.93 ± 0.20	1.70 ± 0.32	2.61 ± 0.46	3.76 ± 0.41
$g_{D_{s0}^{-}{\overset{¯}{D}}^{⁎0}K_{1}^{-}(1400)}$	4.17 ± 0.65	4.09 ± 0.56	3.81 ± 0.45	3.26 ± 0.48	−1.81 ± 0.31

## 4 Conclusion

The study focuses on the strong interactions between scalar charmed and bottom mesons ($B_{0}$, $B_{s0}$, $D_{0}$, and $D_{s0}$) and light axial vector mesons (*A*) and pseudoscalar states (*P*) using the LCSR method. The strong couplings of the physical states $K_{1}^{-}(1270)$ and $K_{1}^{-}(1400)$ are plotted against the mixing angle $\theta_{K}$. These couplings play a crucial role in the study of heavy ion collisions and quarkonium state production. However, both the current manuscript and a previous reference (Ref. [131]) only consider leading-order QCD computations for the correlation functions. Recent studies (Ref. [140] and [141]) have shown that the next-to-leading-order QCD corrections for hadronic and magnetic couplings can have significant impacts on the numerical predictions. Additionally, a comprehensive presentation of the QCD light-cone sum rule approach and its applications in heavy quark physics, along with its technical benefits and theoretical constraints, has been provided in a recent publication (Ref. [139]). These ideas can be incorporated in future papers to enhance the analysis of 3-point couplings using the QCD light-cone sum rule.

## CRediT authorship contribution statement

**S. Momeni:** Writing – review & editing, Writing – original draft, Investigation, Conceptualization. **M. Saghebfar:** Writing – review & editing, Resources, Conceptualization.

## Declaration of Competing Interest

The authors declare that they have no known competing financial interests or personal relationships that could have appeared to influence the work reported in this paper.

## Data Availability

All experimental data used in this study are described in the reference [135]. Also, other considered theoretical data are referenced in the text of each section, and the data from the output of this article are also mentioned in the Table 4, Table 5, Table 6.

## Appendix A Two parton DAs and twist function definitions

In this appendix, the definitions for the twist functions are presented. The approximate forms of the twist-2 functions, for $A=a_{1}$ and $K_{1A}$ states, are as [133]:

$$\Phi_{∥}(u)=6u\overset{¯}{u}\left[1+3a_{1}^{∥}\;\xi +a_{2}^{∥}\;\frac{3}{2}(5\xi^{2}-1)\right],\Phi_{⊥}(u)=6u\overset{¯}{u}\left[a_{0}^{⊥}+3a_{1}^{⊥}\;\xi +a_{2}^{⊥}\;\frac{3}{2}(5\xi^{2}-1)\right],$$

and, for $A=b_{1}$ and $K_{1B}$ are as:

$$\Phi_{∥}(u)=6u\overset{¯}{u}\left[a_{0}^{∥}+3a_{1}^{∥}\;\xi +a_{2}^{∥}\;\frac{3}{2}(5\xi^{2}-1)\right],\Phi_{⊥}(u)=6u\overset{¯}{u}\left[1+3a_{1}^{⊥}\;\xi +a_{2}^{⊥}\;\frac{3}{2}(5\xi^{2}-1)\right],$$

where, ξ=2u−1 and $\overset{¯}{u}=1-u$. $a_{i}^{∥}$ and $a_{i}^{⊥}$ (i=0,1,2) are defined as the Gegenbauer moments of $\Phi_{∥}$ and $\Phi_{⊥}$, respectively. For the relevant two-parton twist-3 chiral-even distribution amplitudes of $A=a_{1}$ and $K_{1A}$, we take the approximate expressions up to conformal spin 9/2 and $O(m_{s})$ as:

$$g_{⊥}^{\left(a\right)}(u)=\frac{3}{4}(1+\xi^{2})+\frac{3}{2}\;a_{1}^{∥}\;\xi^{3}+\left(\frac{3}{7}\;a_{2}^{∥}+5\zeta_{3A}^{V}\right)\left(3\xi^{2}-1\right)+\left(\frac{9}{112}\;a_{2}^{∥}+\frac{105}{16}\;\zeta_{3A}^{A}-\frac{15}{64}\;\zeta_{3A}^{V}\omega_{A}^{V}\right)\left(35\xi^{4}-30\xi^{2}+3\right)+5[\frac{21}{4}\zeta_{3A}^{V}\sigma_{A}^{V}+\zeta_{3A}^{A}(\lambda_{A}^{A}-\frac{3}{16}\sigma_{A}^{A})]\xi (5\xi^{2}-3)-\frac{9}{2}a_{1}^{⊥}\;{\overset{˜}{\delta }}_{+}\;\left(\frac{3}{2}+\frac{3}{2}\xi^{2}+\ln u+\ln\overset{¯}{u}\right)-\frac{9}{2}a_{1}^{⊥}\;{\overset{˜}{\delta }}_{-}\;(3\xi +\ln\overset{¯}{u}-\ln u),$$

$$g_{⊥}^{\left(v\right)}(u)=6u\overset{¯}{u}\{1+(a_{1}^{∥}+\frac{20}{3}\zeta_{3A}^{A}\lambda_{A}^{A})\xi +[\frac{1}{4}a_{2}^{∥}+\frac{5}{3}\;\zeta_{3A}^{V}\left(1-\frac{3}{16}\;\omega_{A}^{V}\right)+\frac{35}{4}\zeta_{3A}^{A}](5\xi^{2}-1)+\frac{35}{4}(\zeta_{3A}^{V}\sigma_{A}^{V}-\frac{1}{28}\zeta_{3A}^{A}\sigma_{A}^{A})\xi (7\xi^{2}-3)\}-18\;a_{1}^{⊥}{\overset{˜}{\delta }}_{+}\;(3u\overset{¯}{u}+\overset{¯}{u}\ln\overset{¯}{u}+u\ln u)-18\;a_{1}^{⊥}{\overset{˜}{\delta }}_{-}\;(u\overset{¯}{u}\xi +\overset{¯}{u}\ln\overset{¯}{u}-u\ln u),$$

$$h_{∥}^{\left(t\right)}(u)=3\;a_{0}^{⊥}\xi^{2}+\frac{3}{2}\;a_{1}^{⊥}\;\xi (3\xi^{2}-1)+\frac{3}{2}[a_{2}^{⊥}\xi +\zeta_{3,A}^{⊥}(5-\frac{\omega_{A}^{⊥}}{2})]\;\xi \;(5\xi^{2}-3)+\frac{35}{4}\zeta_{3A}^{⊥}\sigma_{A}^{⊥}(35\xi^{4}-30\xi^{2}+3)+18{\overset{¯}{a}}_{2}^{∥}[\delta_{+}\xi -\frac{5}{8}\delta_{-}(3\xi^{2}-1)]-\frac{3}{2}\;(\delta_{+}\;\xi [2+\ln(\overset{¯}{u}u)]+\;\delta_{-}\;[1+\xi \ln(\overset{¯}{u}/u)])(1+6{\overset{¯}{a}}_{2}^{∥}),$$

$$h_{∥}^{\left(p\right)}(u)=6u\overset{¯}{u}\{a_{0}^{⊥}+[a_{1}^{⊥}+5\zeta_{3A}^{⊥}(1-\frac{1}{40}(7\xi^{2}-3)\omega_{A}^{⊥})]\xi \;\;\;\;\;\;\;\;+(\frac{1}{4}a_{2}^{⊥}+\frac{35}{6}\zeta_{3A}^{⊥}\sigma_{A}^{⊥})(5\xi^{2}-1)-5\;a_{2}^{∥}[\delta_{+}\xi +\frac{3}{2}\delta_{-}(1-\overset{¯}{u}u)]\}-3[\;\delta_{+}\;(\overset{¯}{u}\ln\overset{¯}{u}-u\ln u)+\;\delta_{-}\;(u\overset{¯}{u}+\overset{¯}{u}\ln\overset{¯}{u}+u\ln u)](1+6a_{2}^{∥}),$$

where,

$${\overset{˜}{\delta }}_{\pm }=\frac{f_{A}^{⊥}}{f_{A}}\frac{m_{q_{2}}\pm m_{q_{1}}}{m_{A}},\;\zeta_{3A}^{V(A)}=\frac{f_{3A}^{V(A)}}{f_{A}m_{A}}.$$

Same as the above quantities but for $A=b_{1}$ and $K_{1B}$ states are given as follows:

$$g_{⊥}^{\left(a\right)}(u)=\frac{3}{4}a_{0}^{∥}(1+\xi^{2})+\frac{3}{2}\;a_{1}^{∥}\;\xi^{3}+5\left[\frac{21}{4}\;\zeta_{3,A}^{V}+\zeta_{3,A}^{A}(1-\frac{3}{16}\omega_{A}^{A})\right]\xi \left(5\xi^{2}-3\right)+\frac{3}{16}\;a_{2}^{∥}\left(15\xi^{4}-6\xi^{2}-1\right)+5\;\zeta_{3,A}^{V}\lambda_{A}^{V}\left(3\xi^{2}-1\right)+\frac{105}{16}\left(\zeta_{3,A}^{A}\sigma_{A}^{A}-\frac{1}{28}\zeta_{A}^{V}\sigma_{A}^{V}\right)\left(35\xi^{4}-30\xi^{2}+3\right)-15{\overset{¯}{a}}_{2}^{⊥}[{\overset{˜}{\delta }}_{+}\xi^{3}+\frac{1}{2}{\overset{˜}{\delta }}_{-}(3\xi^{2}-1)]-\frac{3}{2}\;[{\overset{˜}{\delta }}_{+}\;(2\xi +\ln\overset{¯}{u}-\ln u)+\;{\overset{˜}{\delta }}_{-}\;(2+\ln u+\ln\overset{¯}{u})](1+6{\overset{¯}{a}}_{2}^{⊥}),$$

$$g_{⊥}^{\left(v\right)}(u)=6u\overset{¯}{u}\{a_{0}^{∥}+a_{1}^{∥}\xi +[\frac{1}{4}a_{2}^{∥}+\frac{5}{3}\zeta_{3,A}^{V}(\lambda_{A}^{V}-\frac{3}{16}\sigma_{A}^{V})+\frac{35}{4}\zeta_{3,A}^{A}\sigma_{A}^{A}](5\xi^{2}-1)+\frac{20}{3}\;\xi \left[\zeta_{3,A}^{A}+\frac{21}{16}(\zeta_{3,A}^{V}-\frac{1}{28}\;\zeta_{3,A}^{A}\omega_{A}^{A})(7\xi^{2}-3)\right]-5\;{\overset{¯}{a}}_{2}^{⊥}[2{\overset{˜}{\delta }}_{+}\xi +{\overset{˜}{\delta }}_{-}(1+\xi^{2})]\}-6[\;{\overset{˜}{\delta }}_{+}\;(\overset{¯}{u}\ln\overset{¯}{u}-u\ln u)+\;{\overset{˜}{\delta }}_{-}\;(2u\overset{¯}{u}+\overset{¯}{u}\ln\overset{¯}{u}+u\ln u)](1+6{\overset{¯}{a}}_{2}^{⊥}),$$

$$h_{∥}^{\left(t\right)}(u)=3\xi^{2}+\frac{3}{2}\;a_{1}^{⊥}\;\xi (3\xi^{2}-1)+[\frac{3}{2}a_{2}^{⊥}\;\xi +\frac{15}{2}\zeta_{3,A}^{⊥}(\lambda_{A}^{⊥}-\frac{1}{10}\sigma_{A}^{⊥})]\;\xi (5\xi^{2}-3)+\frac{35}{4}\zeta_{3,A}^{⊥}(35\xi^{4}-30\xi^{2}+3)+\frac{9}{2}{\overset{¯}{a}}_{1}^{∥}\;\xi [\delta_{+}\;(\ln u-\ln\overset{¯}{u}-3\xi )-\delta_{-}\;(\ln u+\ln\overset{¯}{u}+\frac{8}{3})]\;,$$

$$h_{∥}^{\left(p\right)}(u)=6u\overset{¯}{u}\{1+a_{1}^{⊥}\xi +\left(\frac{1}{4}a_{2}^{⊥}+\frac{35}{6}\;\zeta_{3,A}^{⊥}\right)(5\xi^{2}-1)+5\zeta_{3,A}^{⊥}[\lambda_{A}^{⊥}-\frac{1}{40}(7\xi^{3}-3)\sigma_{A}^{⊥}]\;\xi \}-9{\overset{¯}{a}}_{1}^{∥}\;\delta_{+}\;(3u\overset{¯}{u}+\overset{¯}{u}\ln\overset{¯}{u}+u\ln u)-9{\overset{¯}{a}}_{1}^{∥}\;\delta_{-}\;(\frac{2}{3}\xi u\overset{¯}{u}+\overset{¯}{u}\ln\overset{¯}{u}-u\ln u)\;.$$

The twist-2, $\varphi_{P}(u)$, distribution amplitude for pseudoscalar meson (*P*) is expanded in a series of Gegenbauer polynomials [138]:

$$\phi_{P}(u,\mu^{2})=6u(1-u)\left(1+\sum_{n=1}^{\infty }a_{n}^{P}(\mu^{2})C_{n}^{3/2}(\xi )\right),$$

where,

$$\rho_{+}^{P}=\frac{{\left(m_{q}+m_{\overset{¯}{q}}\right)}^{2}}{m_{P}^{2}},\;\rho_{-}^{P}=\frac{\left|m_{q}^{2}-m_{\overset{¯}{q}}^{2}\right|}{m_{P}^{2}}\;;$$

and,

(47) $$\phi^{p}(u)=1+3\rho_{+}^{P}(1+6a_{2})-9\rho_{-}^{P}a_{1}+C_{1}^{1/2}(\xi )\left[\frac{27}{2}\;\rho_{+}^{P}a_{1}-\rho_{-}^{P}\left(\frac{3}{2}+27a_{2}\right)\right]+C_{2}^{1/2}(\xi )\left(30\eta_{3P}+15\rho_{+}^{P}a_{2}-3\rho_{-}^{P}a_{1}\right)+C_{3}^{1/2}(\xi )\left(10\eta_{3P}\lambda_{3P}-\frac{9}{2}\;\rho_{-}^{P}a_{2}\right)-3\eta_{3P}\;\omega_{3P}\;C_{4}^{1/2}(\xi )+\frac{3}{2}\;(\rho_{+}^{P}+\rho_{-}^{P})(1-3\;a_{1}+6\;a_{2})\ln u+\frac{3}{2}\;(\rho_{+}^{P}-\rho_{-}^{P})(1+3\;a_{1}+6\;a_{2})\ln\overset{¯}{u},$$

(48) $$\phi^{\sigma }(u)=6u\;\overset{¯}{u}\left[1+\frac{3}{2}\;\rho_{+}^{P}+15\rho_{+}^{P}a_{2}-\frac{15}{2}\;\rho_{-}^{P}a_{1}+\left(3\rho_{+}^{P}a_{1}-\frac{15}{2}\;\rho_{-}^{P}a_{2}\right)C_{1}^{3/2}(\xi \right)+\left(5\eta_{3P}-\frac{1}{2}\;\eta_{3P}\;\omega_{3P}+\frac{3}{2}\;\rho_{+}^{P}a_{2}\right)C_{2}^{3/2}(\xi )+\eta_{3P}\lambda_{3P}C_{3}^{3/2}(\xi )]+9u\overset{¯}{u}(\rho_{+}^{P}+\rho_{-}^{P})(1-3a_{1}+6a_{2})\ln u+9u\overset{¯}{u}(\rho_{+}^{P}-\rho_{-}^{P})(1+3a_{1}+6a_{2})\ln\overset{¯}{u}\;,g_{2}(u)=\frac{10}{3}\;\delta_{P}^{2}\;\xi \;u\;\overset{¯}{u}$$
